# Generation of an artificially attenuated fowl adenovirus 4 viral vector using the reverse genetics system based on full-length infectious clone

**DOI:** 10.1186/s13567-025-01496-x

**Published:** 2025-03-22

**Authors:** Zhihui Tang, Dengfei Feng, Wentao Fan, Liping Yan, Suquan Song

**Affiliations:** 1 MOE Joint International Research Laboratory of Animal Health and Food Safety, College of Veterinary Medicine, Nanjing Agricultural University, Nanjing, 210095 Jiangsu China; 2 Jiangsu Engineering Laboratory of Animal Immunology, Jiangsu Detection Center of Terrestrial Wildlife Disease, Institute of Immunology, Nanjing Agricultural University, Nanjing, 210095 Jiangsu China

**Keywords:** FAdV-4, infectious clone, reverse genetics system, attenuated viral vector

## Abstract

Fowl adenovirus serotype 4 (FAdV-4) is a non-enveloped double-stranded DNA virus with a 43–45 kb genome. This characteristic makes it a promising viral vector for expressing other antigens in developing multi-valent or multi-series vaccines in the poultry industry. To create an easy-to-use reverse genetics system for manipulating FAdV-4 genomic DNA, a full-length infectious clone of FAdV-4 was constructed using lambda Red-mediated recombination in *Escherichia coli* DH10B. Viable viruses were successfully rescued after the transfection of linearised infectious clones into LMH cells. The rescued viruses showed the same cytopathic effect and growth kinetics as wild-type FAdV-4 viruses. Based on the FAdV-4 infectious clone, the hexon coding sequence of the high-pathogenicity FAdV-4 was replaced by that of the nonpathogenic FAdV-4 using lambda Red-mediated recombination combined with *rpsL* counter selection without leaving extra sequences after engineering. The rescued recombinant virus was highly attenuated and showed low pathogenicity to 21-day-old SPF chickens. Hereto, the easy-to-use reverse genetics system for FAdV-4 was successfully established. With this platform, the genomic DNA of FAdV-4 can be manipulated and purified in DH10B, making it quicker and easier to generate a recombinant FAdV-4 virus to develop multi-valent/multi-series vaccines.

**Supplementary Information:**

The online version contains supplementary material available at 10.1186/s13567-025-01496-x.

## Introduction

High-pathogenicity fowl adenovirus serotype 4 (FAdV-4) is the pathogen responsible for hepatitis and hydropericardium syndrome [1]. Since 2015, this virus has spread widely in China, leading to significant economic losses [2]. FAdV-4 is a non-enveloped double-strand DNA virus with a genome length of 43–45 kb [3] and can infect various animals [4, 5]. Most FAdV-4 strains isolated in China exhibit a 1961-bp nucleotide deletion in the 3’ region of the genome [6]. Although this deleted sequence is not associated with the increased virulence of the newly emerged FAdV-4 in China [7, 8], it creates a site for inserting exogenous sequences [9]. This characteristic makes FAdV-4 a potential viral vector for developing multi-valent/multi-series vaccines. Furthermore, some open reading frames within the FAdV-4 genome have been identified as non-essential genes and can be deleted or replaced by other sequences [10, 11], enhancing the genome’s capacity for foreign gene insertion. There have been efforts to express foreign proteins using FAdV-4 [9, 12–14].

The manipulation of long DNA sequences, such as FAdV-4 genomic DNA, in vitro, is complex. This is because limited restriction enzymes are available, and PCR techniques often struggle to amplify such long sequences. Typically, after virus infection, FAdV-4 genomic DNA can be engineered through homologous recombination using donor DNA in eukaryotic cells. However, obtaining a recombinant virus can be challenging due to the low efficiency of naturally occurring recombination.

The CRISPR-Cas system can enhance this process by introducing double-strand breaks in the DNA through the action of guide RNA and the Cas protein. This triggers endogenous DNA repair processes, improving recombination efficiency in vivo [15]. Nevertheless, even with this approach, purifying recombinant viruses requires multiple rounds of limiting dilution assays, which are both expensive and time-consuming [8, 16].

A cheaper and easier method involves generating an infectious clone of FAdV-4 and manipulating it in bacteria. After cloning the genomic DNA into a backbone plasmid, FAdV-4 DNA can be stored in bacteria and efficiently modified using lambda Red-mediated recombination [7, 11, 17]. The recombinant virus can then be generated by transfecting the engineered FAdV-4 genomic DNA into cells. Additionally, modified infectious clones have been identified and purified as a single genotype, eliminating the need for limiting dilution assays after virus rescue.

The lambda Red-mediated recombination system comprises three functional proteins: Exo, Beta, Gam. Exo degrades linear double-stranded DNA (dsDNA) from the 5’ end, generating a partially dsDNA duplex with single-stranded DNA (ssDNA) overhangs on both ends. Beta binds to these exposed 3’ overhangs, facilitating their annealing to a complementary ssDNA target within the cell. Meanwhile, Gam inhibits the endogenous RecBCD and SbcCD nucleases from digesting the linear DNA introduced into the bacteria [17].

The S12 ribosomal protein coding by the *rpsL* gene is the target of streptomycin. Mutations in the *rpsL* gene confer resistance to streptomycin in bacteria such as DH10B and TOP10 [18]. This characteristic allows the *rpsL* gene to be utilised as a counter-selection marker in DNA engineering [19].

To enhance the study of FAdV-4's genomic functions and develop it into a viral vector, this research constructed an infectious clone containing the full-length FAdV-4 genomic DNA using lambda Red-mediated recombination in *Escherichia coli* DH10B. By integrating an rpsL counter-selection system, an effective and user-friendly reverse genetics system for FAdV-4 was successfully established. This new platform allows for the rapid generation of recombinant FAdV-4 viruses at low cost.

## Materials and methods

### Cells and plasmids

Wild-type FAdV-4 isolate CH_JS_2017 (wtFAdV-4, GenBank: OR584077.1) was isolated, identified, and stored in our laboratory [20]. The viruses used in this study were propagated in the Leghorn Male Hepatoma cell line (LMH, ATCC CRL-2117) and cultured in Dulbecco’s Modified Eagle Medium/Nutrient Mixture F-12 (DMEM/F12, Gibco, USA) containing 10% fetal bovine serum (FBS, Gibco, Australia) at 37 °C with 5% CO_2_. The backbone plasmids pACYC177 and pBeloBAC11 (Fenghui Biotech, China) were stored and amplified in DH10B (Biomed, China). The plasmid pRedET was included in the Counter Selection BAC modification kit (Gene Bridges, Germany). All plasmids were purified using the TIANprep Mini Plasmid Kit (Tiangen Biotech, China).

### Preparation of FAdV-4 genomic DNA

The median tissue culture infective dose (TCID_50_) of the wtFAdV-4 virus was measured and calculated using the Reed and Muench method [21]. The virus was then introduced into an LMH cell culture at a multiplicity of infection (MOI) of 0.01. Progeny viruses were harvested when more than 80% of the cells exhibited a cytopathic effect (CPE). The genomic DNA of wtFAdV-4 was extracted using an animal genomic DNA extraction kit (Tsingke Biotech, China) in accordance with the manufacturer’s instructions. The purified DNA was verified by running the sample on a 20-cm long, 1% standard agarose gel at 30 V overnight, and its concentration was subsequently determined.

### Polymerase chain reaction (PCR)

Table 1 presents the primers for the construction and identification of the plasmids. Sangon Biotech, China, synthesised all primers. For high-fidelity PCR, the amplification of fragments for plasmids construction was performed in a final volume of 50 μL using 1 × Hieff Canace® Plus PCR Master Mix (Yeasen Biotech, China) and 20 pmol of each used primer. For regular PCR, which was used to identify positive clones, the reactions were carried out in a final volume of 25 μL using 1 × Hieff® PCR Master Mix and 10 pmol of each primer.

**Table 1:** **Lists of primers**

Primer Name	Primer sequence (5' → 3')	Product (bp)	Purpose
pA-delTn903 F	TGATG***ttaattaa***CTCAGTATTGCCCGCTCCA	1979	Constructe the infectious clone backbone
pA-delTn903 R	TGATG***ttaattaa***TTCCGCTTCCTCGCTCACT		
5' HR4K F	AGCGGAA***ttaattaa***CATCATCTTATATAACCGCGTCT	4067	Generate a 4 kb fragment in the 5' region of FAdV-4 genome with *Pac*I and *Hind* III restriction sites
5' HR4K R	GGTAATGTTG***aagctt***CTACCCAACGCACATACACACT		
3' HR4K F	CGTTGGGTAG***aagctt***CAACATTACCCACCGCACACT	4095	Generate a 4 kb fragment in the 3' region of FAdV-4 genome with *Pac*I and *Hind* III restriction sites
3' HR4K R	TACTGAG***ttaattaa***CATCATCTTATATAACCGCGTCT		
idHR4K F	CTCGTGAGGACTTGGAATGAC	432	Identify the p15A-AmpR-HR4K plasmid
idHR4K R	CGTGACCGAAACCGCTGATT		
FAdV4 403F	ATACCAACACGAGCACCTC	403	Identify the p15A-AmpR-FAdV4 plasmid (infectious clone)
FAdV4 403R	TTATCCCTGAACCCGATG		
DHexon_rL F	AACCCGCTGGCTCCCAAGGAGTCCATGTTTAACAACTGGTCGGAGACGGCGGCCTGGTGATGATGGCGGGATCG	1419	Generate the recombinant plasmid pDHexon_rL
DHexon_rL R	GTGTCGAACACGCCATAGAGCATGTACACGTAAGTGGGATCATCCATGGGTCAGAAGAACTCGTCAAGAAGGCG		
BfON1Hexon F	CGCCCGACCTGACTACCG	435	Generate the 5'-homology arm of ON1 Hexon donor DNA
BfON1Hexon R	CGGACACGTTCTGCCCGGGCGCCGTCTCCGACCAGTTG		
ON1 Hexon F	GCCCGGGCAGAACGTGTCCG	2254	Generate the ON1 Hexon donor DNA
ON1 Hexon R	ATCGAGCTCGAAGTTGATGACCATGC		
AfON1Hexon F	CAGCATGGTCATCAACTTCGAGCTCGATCCCATGGATGATCCCACT	418	Generate the 3'-homology arm of ON1 Hexon donor DNA
AfON1Hexon R	GTTTCTTGTCGGACCACC		
pBAC F	CGGTTGTAAGTGTGTCAAAAGACGCGGTTATATAAGATGATG***ttaattaa***CGTCGACAGCGACACACTTGC	6626	Generate the MiniF-CmR with 50 bp homology arms of pFAdV4 in both 5'- and 3'-side
pBAC R	CGGTTGTAAGTGTGTCAAAAGACGCGGTTATATAAGATGATG***ttaattaa***GCCTGGGGTGCCTAATGAGTG		

Letters in bold italic represent restriction sites, and underlined letters represent homology arms

The PCR conditions were as follows: initial denaturation at 95 °C for 5 min; 30 cycles of denaturation at 95 °C for 10 s; annealing at 60 °C for 15 s, extension at 72 °C for 30 s /kb; and a final extension at 72 °C for 5 min.

### Construction of the FAdV-4 infectious clone

A PCR product containing the p15A origin and β-lactamase expression cassette (AmpR) was amplified with the primer pairs pA-delTn903 F/R. The 4 kb homologous region for the 5’- and 3’-termination of FAdV-4 genomic DNA (5’-HR and 3’-HR) were amplified with the primer pairs 5' HR4K F/R and 3' HR4K F/R. After gel purification, all three PCR products were retrieved using the FastPure DNA Extraction Mini Kit (Vazyme, China). Then, these fragments constructed the plasmid p15A-AmpR-HR4K (pHR4K) at a molar ratio of 1:1:1 using the ClonExpress Ultra One Step Cloning Kit (Vazyme).

Positive clones were identified and verified using the primer pairs idHR4K F/R, restriction enzymes digestion (Takara, Japan), and Sanger sequencing (Sangon Biotech, China). The plasmid pHR4K was digested with *Hin*d III. Subsequently, 1 μg of linearised pHR4K [22] and 500 ng of FAdV-4 genomic DNA were co-electroporated into DH10B containing the plasmid pRedET to construct FAdV-4 infectious clone p15A-AmpR-FAdV4 (pFAdV4). The DH10B competent cells were prepared in accordance with the manufacturer’s instructions of the Counter Selection BAC modification kit. Still, the electroporation conditions were slightly adjusted: 1500 V, 25 μF, 200 Ohms, 1 mm electroporation cuvette (Bio-Rad, USA). Positive clones were further identified and verified using the primer pairs FAdV4 403F/R, restriction enzymes digestion, and Illumina MiSeq sequencing (Sangon Biotech).

### Virus rescue and growth kinetics

2 μg of purified pFAdV4 were linearised by digestion with *Pac* I. The entire mixture was transfected into LMH cells grown in 12-well plates using Hieff Trans® Liposomal Transfection Reagent (Yeasen Biotech). When CPE was observed, the supernatant of the cell culture was harvested after three rounds of freeze–thaw cycles and subsequently propagated. The rescued virus derived from the linearised pFAdV4 was named rFAdV-4 (rescued FAdV-4).

LMH cells were infected with wtFAdV-4 or rFAdV-4 at an MOI of 0.01. The infected cells and supernatants were harvested 12, 24, 36, 48, 60, and 72 h post-infection (hpi). Then, the virus titre of each time point was calculated.

### Sequence identity analysis

The full-genomic sequence of the FAdV-4 CH_JS_2017 strain was aligned with the 19 FAdV-4 reference sequences obtained from the GenBank database at the National Center for Biotechnology Information. The alignment was performed using the MEGA software package (version: 11.0.13). The genetic distances between the sequences were calculated using the maximum composite likelihood method and the bootstrap test of 1000 replicates. Detailed information about reference sequences can be found in Additional file 1.

### Replace CH_JS_2017 hexon with ON1 hexon

Sangon Biotech, China, synthesised the hexon sequence of the nonpathogenic FAdV-4 isolate ON1 (GenBank: GU188428.1) [23]. To better use rpsL counter selection, the backbone of pFAdV4 was replaced with linearised pBeloBAC11, MiniF replicon, and chloramphenicol acetyltransferase (CmR), and the modified infectious clone was named pMiniF-CmR-FAdV4 (pBAC-FAdV4).

Briefly, the MiniF-CmR, which contained 50 bp homology arms of pFAdV4 on both the 5'- and 3'-side was amplified with the primer pairs of pBAC F/R and purified. The retrieved PCR product was then electroporated into *L*-arabinose-induced DH10B competent cells containing pFAdV4 and pRedET. After recombination, positive clones were picked out under the selection pressure of chloramphenicol and validated by restriction enzyme digestion. Then, following the instructions of the Counter Selection BAC modification kit (Gene Bridges), the recombinant infectious clone pBAC-FAdV4-ON1 Hexon (pON1) was constructed using two-round lambda Red-mediated recombination combined with *rpsL* counter selection. The positive clones were verified via Sanger sequencing.

### Animal trials

Forty 21-day-old SPF chickens were randomly divided into four groups (I to IV), with ten chickens in each group. Chickens from groups II to IV were inoculated with 0.3 mL (10^6.5^ TCID_50_/0.1 mL) wtFAdV-4, rFAdV-4, or with the recombinant virus ON1 Hexon (rON1) via intramuscular injection [24]. Chickens from group I were inoculated with 0.3 mL of PBS and served as controls. Clinical signs and survival rates for each group were recorded daily for seven days. At 2 days post-inoculation (dpi), five chickens that died from the treatments in each of the wtFAdV-4 and rFAdV-4 groups were dissected for anatomical pathological observation. At the same time, five chickens from both the rON1 and control groups were euthanised using carbon dioxide asphyxiation and then dissected. The dissected chickens' liver, spleen, and kidney were collected and fixed in 4% neutral formalin buffer for histopathological observation.

### Statistical analysis

All statistical analyses were performed using the GraphPad Prism software package (version: 9.0.0). To analyse the growth curves; a t-test was performed between wtFAdV-4 and rFAdV-4 at each time point. Data were represented as means ± SD. A log-rank test was performed to analyse the survival curves, and the control, rFAdV-4, and rON1 groups were compared with the wtFAdV-4 group separately. *P* values less than 0.05 and 0.01 indicated statistical significance and extreme significance, respectively.

## Results

### Construction of the FAdV-4 infectious clone

**Figure 1:**
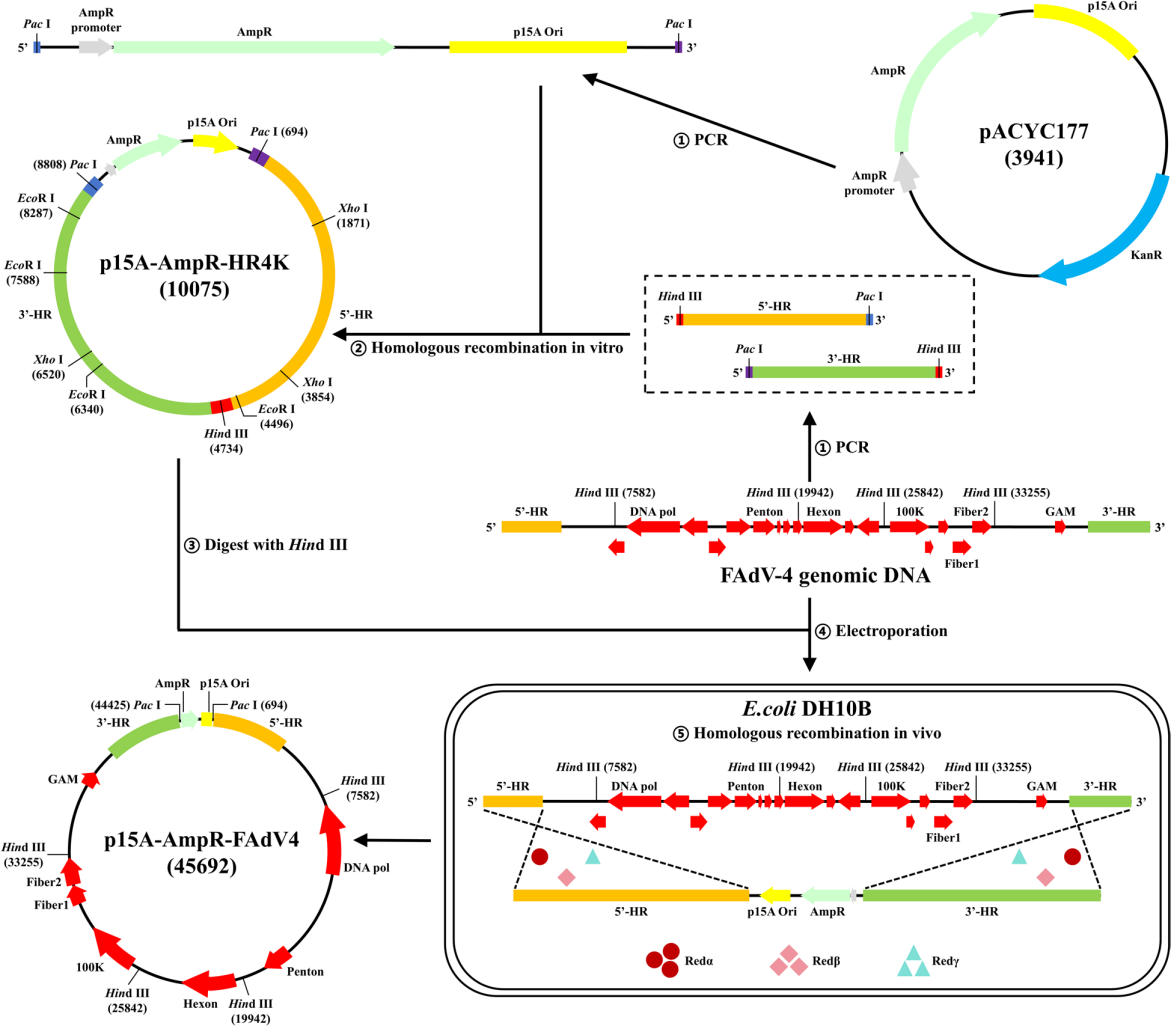
**Schematic diagram illustrating the generation of the FAdV-4 infectious clone**.

An infectious clone was constructed following the schematic diagram in Figure 1 to develop an easy-to-use reverse genetics system for manipulating FAdV-4 genomic DNA.

**Figure 2:**
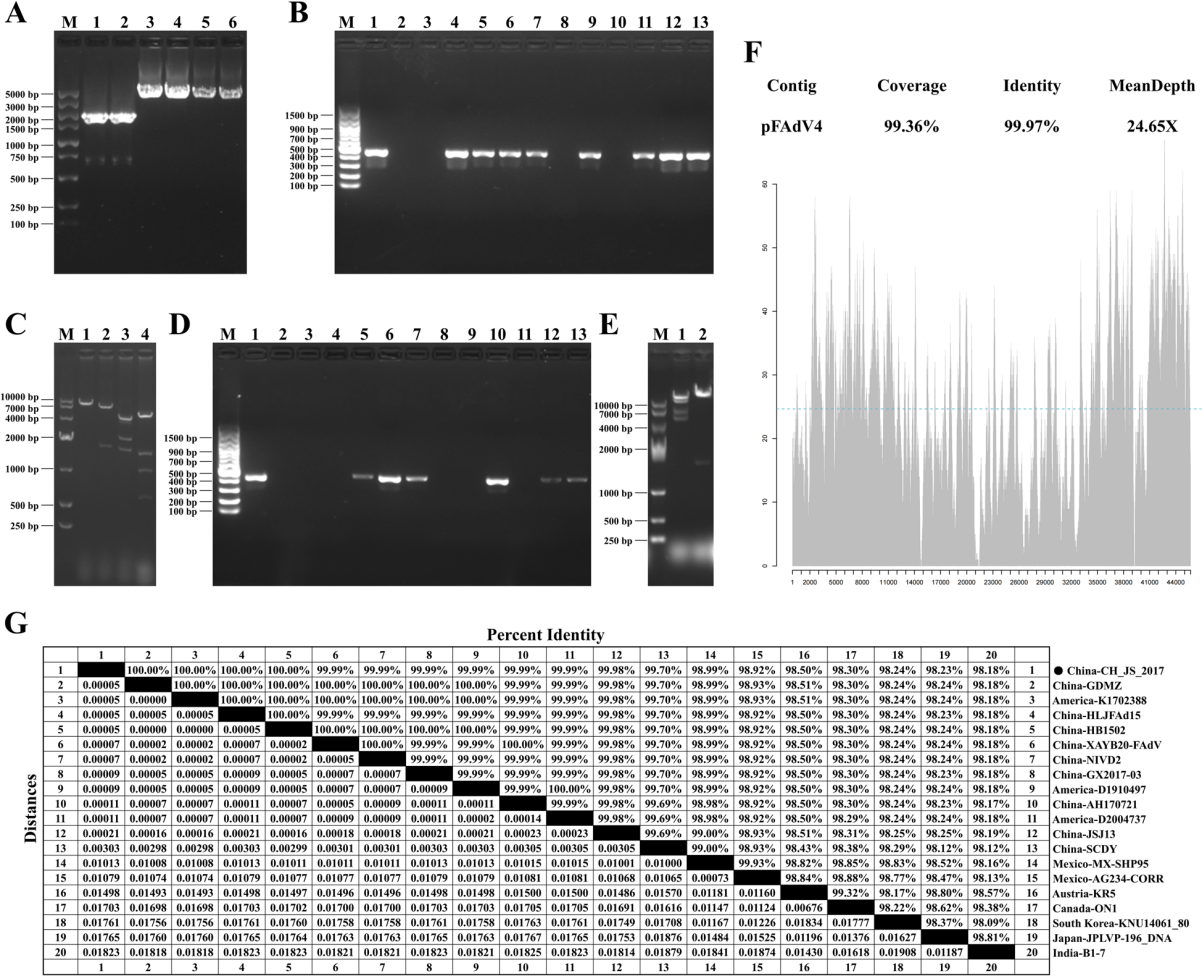
**Construction of the FAdV-4 infectious clone. A** Gel analysis of the PCR products for generating pHR4K. M: marker; 1–2: pA-delTn903 F/R; 3–4: 5' HR4K F/R; 5–6: 3' HR4K F/R. **B** Identification of the pHR4K. M: marker; 1: positive control; 2: negative control; 4/5/6/7/9/11/12/13: clones identified as positive; 3/8/10: clones identified as negative. **C** Restriction enzyme analysis of the pHR4K. M: marker; 1: *Hin*d III; 2: *Pac* I; 3: *Xho* I; 4: *Eco*R I. **D** Identification of the pFAdV4. M: marker; 1: positive control; 2: negative control; 5/6/7/10/12/13: clones identified as positive; 3/4/8/9/11: clones identified as negative. **E** Restriction enzyme analysis of the pFAdV4. M: marker; 1: *Hin*d III; 2: *Pac* I. **F** Sequencing depth map of the pFAdV4 infectious clone. **G** Pairwise distance analysis of full-genome sequences among different FAdV-4 strains.

An intermediate plasmid originating from the plasmid pACYC177 was constructed. As depicted in Figure 2A, the backbone of pACYC177, the FAdV-4 5’-terminal fragment, and the FAdV-4 3’-terminal fragment were amplified and retrieved. These three PCR products were assembled in vitro using a commercial cloning kit and transferred into DH10B. Positive clones were identified under the selection pressure of ampicillin and confirmed by gene-specific amplification. Eight clones were identified as positive (Figure 2B). Three of these clones were further validated through restriction enzyme digestion (Figure 2C, one representative gel image) and Sanger sequencing. The complete image of Figure 2C can be found in Additional file 2.

The homology arms of the FAdV-4 genomic DNA were revealed by digesting pHR4K with *Hin*d III. Linearised pHR4K and purified FAdV-4 genomic DNA were co-electroporated into DH10B competent cells containing pRedET and subsequently induced with *L*-arabinose. Following recombination, the infectious clone of FAdV-4 was successfully constructed. The selection pressure of ampicillin and gene-specific amplification identified positive clones. Six clones were confirmed as positive (Figure 2D), and three of these were further validated through restriction enzyme digestion (Figure 2E, one representative gel image) and Illumina MiSeq sequencing. The analysis revealed that the sequences of the five constructed FAdV-4 infectious clones were identical. The obtained sequence mapped 99.36% of the reference (previously assembled by Sanger sequencing), and the identity between them was found to be 99.97% (Figure 2F). A full image of Figure 2E is provided in Additional file 2.

The analysis of pairwise distances among the full-genome sequences of reference strains revealed that the CH_JS_2017 strain exhibited similarities ranging from 99.70% to 100% with FAdV-4 strains isolated from China and The United States. In contrast, it showed similarities between 98.18% and 98.99% with FAdV-4 strains isolated from Austria, Canada, India, Japan, Mexico, and South Korea (Figure 2G).

### Virus rescue and growth kinetics of the rescued viruses

**Figure 3:**
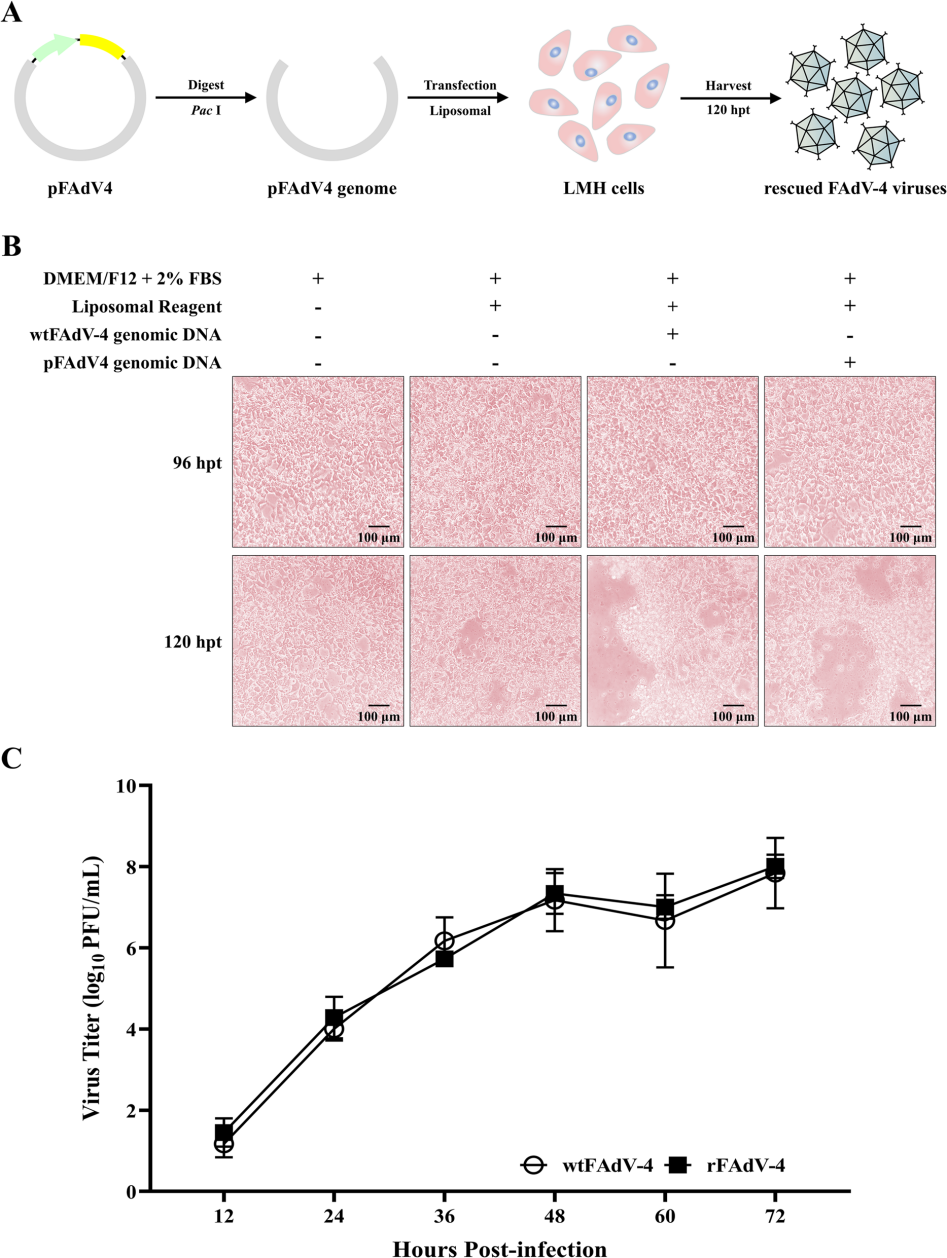
**Virus rescue and the growth kinetics of rescued viruses. A** Schematic diagram illustrating the generation of rescued viruses. **B** CPE was observed around 120 hpt in LMH cells transfected with wtFAdV-4 or pFAdV4 genomic DNA. **C** There was no significant difference (*P* > 0.05) between the growth curves of wtFAdV-4 and rFAdV-4 at each time point.

To verify if viable FAdV-4 viruses could be rescued from the infectious clone, 2 μg of sequence-confirmed infectious clone was linearised by digestion with *Pac*I (pFAdV4 genomic DNA) and then transfected into LMH cells in 12 plates (Figure 3A). The same amount of FAdV-4 genomic DNA purified from wtFAdV-4 was a positive control. At 120 h post-transfection (hpt), both LMH cells transfected with wtFAdV-4 and the pFAdV4 genomic DNA showed CPE (Figure 3B). No CPE occurred in blank cells or cells to which only liposomal reagent was added. The rescued virus was harvested at 120 hpt and inoculated into new LMH cells. After five passages, the growth kinetics of wtFAdV-4 and rFAdV-4 were tested. As shown in Figure 3C, the virus growth curve showed there was no significant difference (*P* > 0.05) between wtFAdV-4 and rFAdV-4 at each time point and it reached 10^7.8^ PFU/mL at 72 hpi.

### Construction of the recombinant infectious clone pON1 Hexon

**Figure 4:**
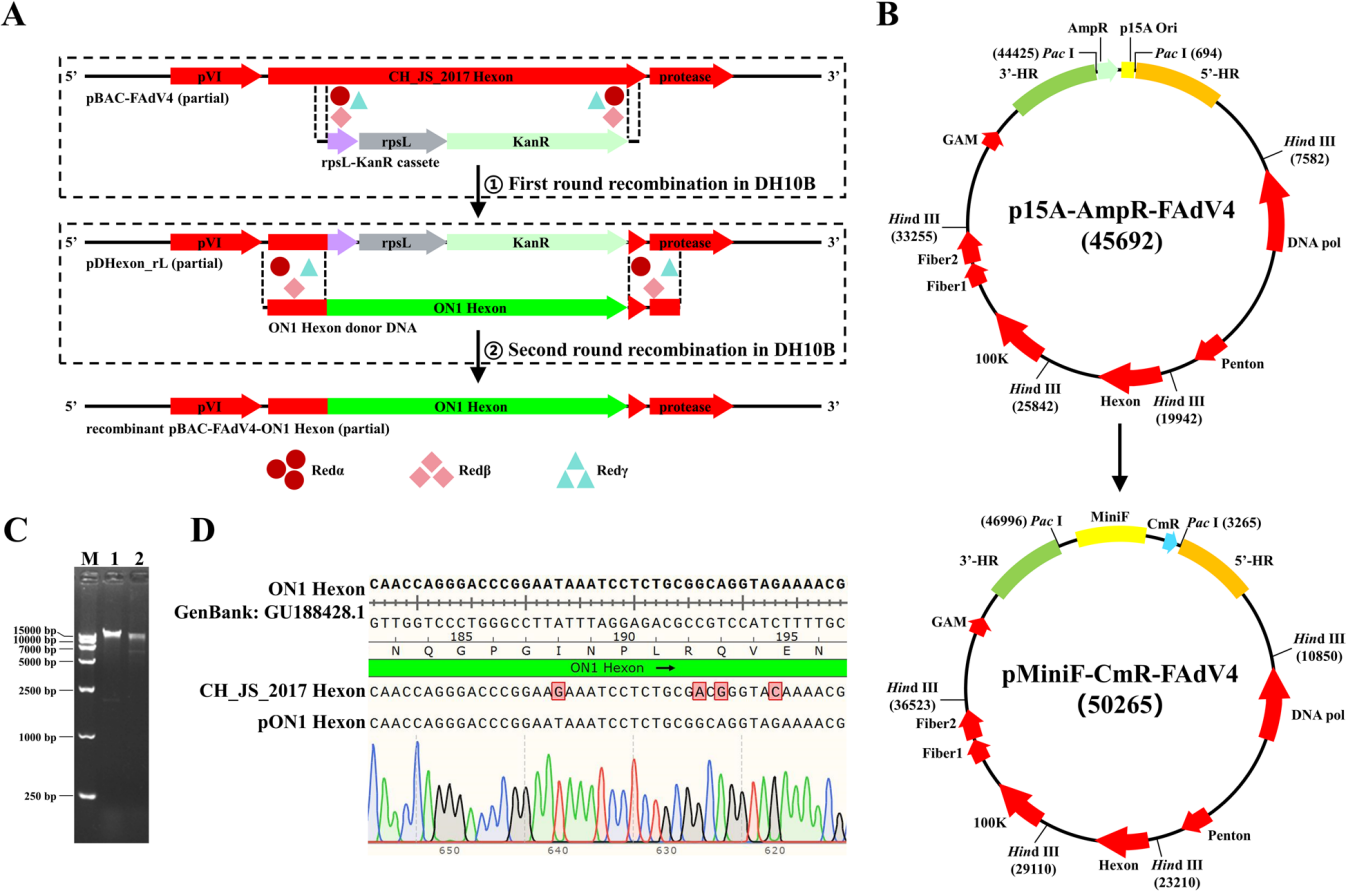
**Construction of the recombinant FAdV-4 infectious clone pON1. A** Schematic diagram illustrating the two-round lambda Red-mediated recombination combined with *rpsL* counter selection. **B** Schematic diagram for the construction of pBAC-FAdV4. **C** Restriction enzyme analysis of pFAdV4 and pBAC-FAdV4. M: marker; 1: pFAdV4 digested with *Pac* I; 2: pBAC-FAdV4 digested with *Pac* I. **D** Sanger sequencing result of pON1.

To construct a recombinant FAdV-4 infectious clone in which the hexon sequence was replaced with that of the ON1 hexon, a two-round lambda Red-mediated recombination method was conducted following the manufacturer’s instructions of the Counter Selection BAC modification kit (Figure 4A). The desired plasmid was not constructed, as there were almost the same number of clones in both induced and uninduced plates, which did not meet the requirements of the manual. For better use of the Counter Selection BAC modification kit, as shown in Figure 4B, the backbone of pFAdV4 (p15A ori-AmpR) was replaced with that of pBeloBac11 (MiniF-CmR, Figure 4C), which was analysed by *Pac* I digestion. Based on pBAC-FAdV4, the hexon coding sequence was successfully replaced with that of the ON1 (Figure 4D, pON1 Hexon).

### Replacement with ON1 hexon attenuates high-pathogenicity FAdV-4

To study the pathogenicity of the recombinant virus rON1, 21-day-old chickens were inoculated with rON1 at 0.3 × 10^6.5^ TCID_50_. Chickens in the wtFAdV-4 and rFAdV-4 groups appeared depressed and were observed huddled together, with ruffled feathers, at 1 dpi. They then passed away at 2 dpi. Nine chickens died in the wtFAdV-4 group, and ten died in the rFAdV-4 group. No clinical signs were observed in the control and rON1 groups. Of the chickens that died from the treatments within the wtFAdV-4 and rFAdV-4 groups, five from each group were dissected.

**Figure 5:**
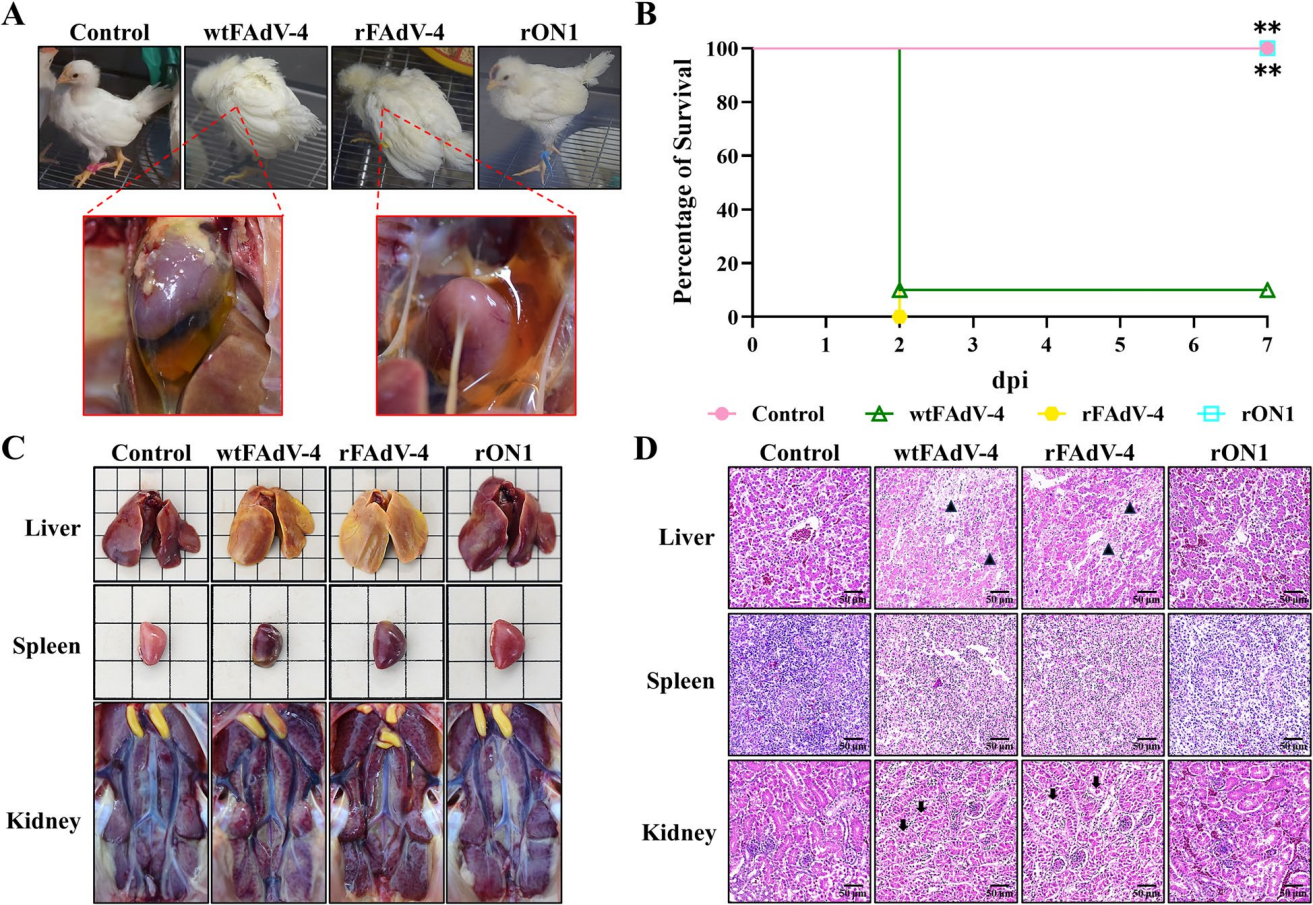
**Replacement with the ON1 Hexon attenuated the high-pathogenicity FAdV-4. A** Clinical sign of each group at 2 dpi and hydropericardium of dead chickens from the wtFAdV-4 and rFAdV-4 groups. **B** Survival rates of each group within 7 days of inoculation (*n* = 10). The control, rFAdV-4, and rON1 groups were compared with the wtFAdV-4 group separately, and the “**” represents *P* < 0.01. **C** Anatomical, pathological changes in the liver, spleen and kidney of chickens from each group at 2 dpi. **D** Histopathological changes in the liver, spleen and kidney of chickens from each group at 2 dpi. The triangle markers indicate areas of hepatocyte necrosis and loss of hepatic cords, while the arrow markers indicate areas with destruction of renal tubular epithelial cells.

Meanwhile, five chickens from both the control and rON1 groups were euthanised by carbon dioxide asphyxiation and then dissected. During necropsy, a large amount of transparent, pale-yellow fluid was observed in the pericardium of deceased chickens from both the wtFAdV-4 and rFAdV-4 groups (Figure 5A).

The survival rates of the wtFAdV-4, rFAdV-4, rON1, and control groups were 10%, 0%, 100%, and 100% (Figure 5B). These results showed that the high-pathogenicity FAdV-4 was attenuated after the hexon gene was replaced with that of the ON1 strain.

Organic injuries were observed in chickens from the wtFAdV-4 and rFAdV-4 groups. These injuries included a yellowish liver, a congested spleen, and a swollen kidney (Figure 5C). Histopathological analysis revealed microscopic tissue lesions in the wtFAdV-4 and rFAdV-4 groups, including massive necrosis of hepatocytes and the disappearance of hepatic cords in the liver, severe depletion of lymphocytes in the spleen, and the destruction of renal tubular epithelial cells (Figure 5D).

In the rON1 group, there was a slight depletion of lymphocytes in the spleen and mild interstitial haemorrhage in the kidney (Figure 5D) despite no visible organ injuries being found at necropsy (Figure 5C). Additional file 3 shows the detailed numbers of birds showing clinical signs, organs with gross lesions, and organs with histopathological changes.

## Discussion

The length of the FAdV-4 genome is approximately 43–45 kb, and few restriction enzyme recognition sites are available for manipulating the FAdV-4 genomic DNA in vitro. Conducting manipulations in eukaryotic cells is challenging due to the low efficiency of natural recombination and the difficulty of purifying the recombinant virus. A more practical approach involves manipulating the infectious clone of FAdV-4 in bacteria. Some infectious clones of different serotypes of FAdVs have been constructed using several methods, which can be categorized as follows: recA-mediated recombination in *E. coli* BJ5183 [12, 25, 26], lambda Red-mediated recombination in *E. coli* DH10B or GB05-dir [7, 27], and Gibson Assembly in vitro [28, 29].

All these methods require that both ends of the vector and the viral genome contain homologous sequences that form 3' ssDNA overhangs, allowing them to anneal with complementary 3' ssDNA overhangs [17, 30, 31]. In this study, the generated infectious clone could be further manipulated by Red/ET. The infectious clone of FAdV-4 CH_JS_2017 was constructed using lambda Red-mediated recombination in DH10B. Although a 50-bp homology arm was sufficient for lambda Red-mediated recombination [17], in this study the FAdV-4 infectious clone was constructed with just the homology arms of 4 kb [12].

Using short homology arms such as 50 bp, only a portion of the ssDNA overhang (not more than 50 bp) could anneal with the target DNA. Reference 12 describes using 4 kb homology arms to provide a longer homologous sequence for annealing with the target DNA, facilitating homologous recombination. Based on the infectious clone, FAdV-4 genomic DNA can be stored in bacteria and manipulated using the highly efficient Red/ET system [17]. After manipulation, recombinant viruses can be easily isolated under selective antibiotic pressure.

In contrast to infectious clones of turkey herpesvirus, duck enteritis virus and porcine pseudorabies virus, where the replicon and selection gene were integrated into the virus genome [32–34], the replicon and selection genes of the FAdV-4 infectious clone were not inserted into the viral genome, allowing for straightforward removal through digestion with *Pac* I. In this study, during virus rescue, 2 μg of the infectious clone plasmid was sufficient for transfection, and the linearised plasmid was directly transfected into LMH cells without requiring further purification of the FAdV-4 genomic DNA to eliminate enzymes and buffers used during digestion. According to the manufacturer’s instructions, the entire cell culture medium should be removed and replaced with a fresh medium within 6–8 h after transfection, ensuring that residual enzymes and buffers do not affect the virus rescue procedure.

Before manipulating the genomic DNA of FAdV-4, we carefully considered which appropriate methods to use. Although the Flp-FRT system is known for its high recombination efficiency and is commonly used for manipulating target sequences in bacteria [34, 35], we decided not to use it for this study. This decision was made because the Flp-FRT system leaves an FRT sequence in the target DNA after recombination, which limits further manipulation of sequences near the existing FRT site. Instead, we opted for a seamless cloning method using *rpsL* counter selection to engineer the FAdV-4 infectious clone.

To prevent undesirable recombination between plasmids within the same bacterium, we utilized the low-copy-number plasmid pACYC177 as the backbone for the infectious clone. However, during the process of generating the recombinant infectious clone pON1, no positive clone could be identified under the pressure of streptomycin after the second-round recombination. This failure may have been due to the fact that the FAdV-4 infectious clone with the p15A origin was a multi-copy plasmid. During the second round of recombination, some plasmids in one bacterium were successfully recombined while others were not. Those plasmids that had not undergone successful recombination still contained the *rpsL* counter-selection marker, which ultimately caused the bacteria with mixed plasmids to die under the pressure of streptomycin.

This problem was resolved by replacing the p15A origin with the MiniF replicon derived from the fertility factor of *E. coli* (one-copy plasmid). Following this change, we successfully constructed the recombinant FAdV-4 infectious clone in which the hexon gene was replaced by that of the ON1.

The pathogenicity of the wtFAdV-4 CH_JS_2017 strain has been investigated, revealing that the survival rate of 21-day-old and 28-day-old chickens inoculated intramuscularly with 10^6.5^ TCID_50_ of wtFAdV-4 ranged from 0 to 10% [20, 24]. These findings indicate that the pathogenicity of wtFAdV-4 is comparable to other highly pathogenic FAdV-4 strains identified in previous studies, including the first study on purified wild-type pathogenic FAdV-4 [36]. Therefore, the observed differences in survival rates between wtFAdV-4 and rFAdV-4 in this study should be attributed to individual animal variation. Although one chick survived in the wtFAdV-4 group by the end of the experiment (7 dpi), it exhibited severe clinical signs by 2 dpi.

The pathogenicity and replication ability of FAdV-4 is influenced by several factors [1], including the cellular damage induced by both structural and non-structural viral proteins [7, 37–39], the age and genetic background of the host [5, 40], host proteins that regulate the viral life cycle [41, 42], variations in the inoculation route and dosage [43, 44], and co-infection with other pathogens [45, 46]. Using a user-friendly reverse genetics system, the high-pathogenicity FAdV-4 strain could be artificially attenuated through genome editing. In this study, the hexon gene of the high-pathogenicity FAdV-4 CH_JS_2017 strain was successfully replaced with that of the ON1 strain using lambda Red-mediated two-round recombination combined with *rpsL* counter selection. The rescued recombinant virus, rON1, demonstrated low pathogenicity in 21-day-old SPF chickens.

While artificially attenuated FAdV-4 presents a safer option for developing live attenuated vaccines, mild histopathological changes were still observed in the liver, spleen, and kidney. Therefore, further studies are necessary to assess the safety of the artificially attenuated FAdV-4 before it can be approved for use in poultry vaccines.

In summary, a user friendly reverse genetics system for FAdV-4, based on an infectious clone, has been successfully established. FAdV-4 genomic DNA can be easily manipulated in bacteria using lambda Red-mediated recombination along with the *rpsL* counter-selection system. These advancements open up possibilities for developing FAdV-4 as a potential viral vector.

## Supplementary Information

**Additional file 1. Detailed information about reference sequences.**

**Additional file 2. Gel electrophoresis image of the restriction enzyme analysis of pHR4K and pFAdV4. A** Gel electrophoresis image of the restriction enzyme analysis of pHR4K. “Column” means plasmids were purified by spin column; “Isopropanol” means plasmids were purified by isopropanol precipitation. **B** Gel electrophoresis image of the restriction enzyme analysis of pFAdV4 and pRed.

**Additional file 3. Number of birds showing clinical signs, organs with gross lesions, and organs with histopathological changes.**

## Data Availability

All data generated or analysed during this study are included in this published article and its supplementary information files.
