# Supplementary material for: Generation of an artificially attenuated fowl adenovirus 4 viral vector using the reverse genetics system based on full-length infectious clone
Source: Vet Res. 2025 Mar 22;56:62. doi: 10.1186/s13567-025-01496-x (PMC11929364; doi:10.1186/s13567-025-01496-x)
Supplement: Supplementary file 1 — Additional file 1. Detailed information about reference sequences. [file 13567_2025_1496_MOESM1_ESM.docx]

**Additional file 1 Detailed information about reference sequences.**

| **Strain** | **Genome (bp)** | **Host** | **Country** | **Isolation Year** | **Accession Number** |
| --- | --- | --- | --- | --- | --- |
| JSJ13 | 43755 | chicken | China | 2013 | KM096544.1 |
| HLJFAd15 | 43720 | chicken | China | 2015 | KU991797.1 |
| HB1502 | 43621 | chicken | China | 2015 | KX421401.2 |
| GDMZ | 43723 | chicken | China | 2016 | MG856954.1 |
| NIVD2 | 43719 | chicken | China | 2017 | MG547384.1 |
| CH_JS_2017 | 43723 | chicken | China | 2017 | OR584077.1 |
| GX2017-03 | 43723 | chicken | China | 2017 | MN577979.1 |
| AH170721 | 43723 | duck | China | 2017 | MW699358.1 |
| SCDY | 43677 | chicken | China | 2018 | MK629523.1 |
| XAYB20-FAdV | 43721 | pigeon | China | 2023 | PP934606.1 |
| JP/LVP-1/96 DNA | 45688 | chicken | Japan | unknown | LC628937.1 |
| KNU14061_80 | 45644 | chicken | South Korea | 2014 | OR352898.1 |
| B1-7 | 45622 | chicken | India | 2011 | KU342001.1 |
| KR5 | 45810 |  | Austria | unknown | HE608152.1 |
| K1702388 | 43723 | chicken | America | 2017 | MW711379.1 |
| D1910497 | 43717 | chicken | America | 2019 | MW711380.1 |
| D2004737 | 43717 | chicken | America | 2020 | MT813039.1 |
| ON1 | 45667 | chicken | Canada | 2004 | GU188428.1 |
| MX-SHP95 | 45641 | chicken | Mexico | 1995 | KP295475.1 |
| AG234-CORR | 45505 | chicken | Mexico | 1995 | MK572849.1 |
