# Supplementary material for: Generation of an artificially attenuated fowl adenovirus 4 viral vector using the reverse genetics system based on full-length infectious clone
Source: Vet Res. 2025 Mar 22;56:62. doi: 10.1186/s13567-025-01496-x (PMC11929364; doi:10.1186/s13567-025-01496-x)
Supplement: Supplementary file 2 — Additional file 2. Gel electrophoresis image of the restriction enzyme analysis of pHR4K and pFAdV4. A Gel electrophoresis image of the restriction enzyme analysis of pHR4K. “Column” means plasmids were purified by spin column; “Isopropanol” means plasmids were purified by isopropanol precipitation. B Gel electrophoresis image of the restriction enzyme analysis of pFAdV4 and pRed. [file 13567_2025_1496_MOESM2_ESM.pptx]

## Slide 1
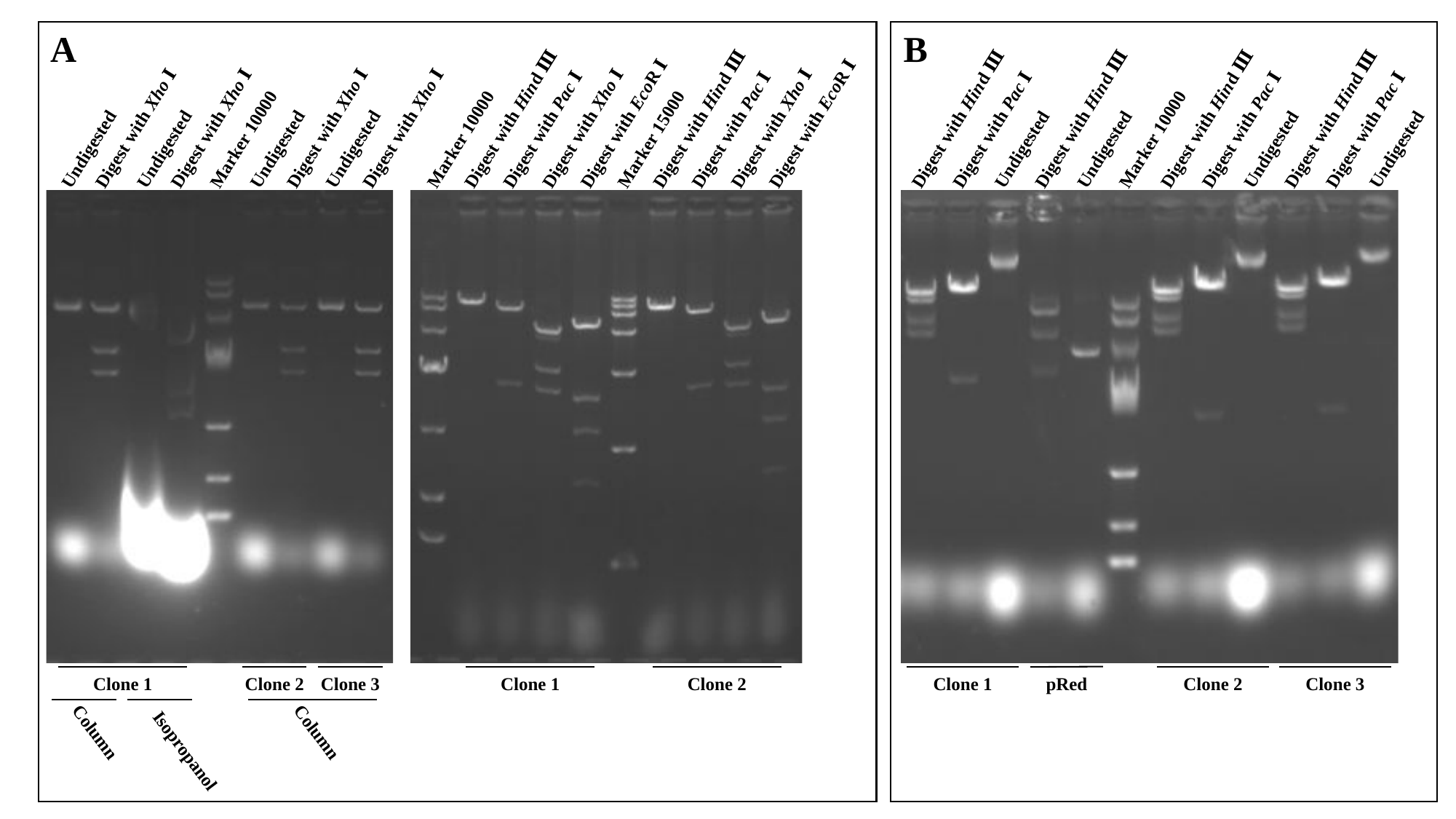

Digest with Hind Ⅲ
Digest with Hind Ⅲ
Digest with Pac Ⅰ
Digest with Hind Ⅲ
Digest with Hind Ⅲ
Digest with Pac Ⅰ
Digest with Pac Ⅰ
Undigested
Undigested
Marker 10000
Undigested
Undigested
pRed
Clone 1
Clone 2
Clone 3
B
Digest with Pac Ⅰ
Digest with Hind Ⅲ
Digest with Hind Ⅲ
Digest with Pac Ⅰ
Digest with EcoR Ⅰ
Digest with EcoR Ⅰ
Marker 10000
Marker 15000
Digest with Xho Ⅰ
Digest with Xho Ⅰ
Clone 1
Clone 2
Digest with Xho Ⅰ
Digest with Xho Ⅰ
Digest with Xho Ⅰ
Digest with Xho Ⅰ
Undigested
Undigested
Marker 10000
Undigested
Undigested
Clone 2
Clone 3
Clone 1
Column
Isopropanol
Column
A
