# Supplementary material for: Generation of an artificially attenuated fowl adenovirus 4 viral vector using the reverse genetics system based on full-length infectious clone
Source: Vet Res. 2025 Mar 22;56:62. doi: 10.1186/s13567-025-01496-x (PMC11929364; doi:10.1186/s13567-025-01496-x)
Supplement: Supplementary file 3 — Additional file 3. Number of birds showing clinical signs, organs with gross lesions, and organs with histopathological changes. [file 13567_2025_1496_MOESM3_ESM.docx]

**Additional file 3 Number of birds showing clinical signs, organs with gross lesions, and organs with histopathological changes.**

| **Number of the birds showed clinical signs** | | | | | | | | |
| --- | --- | --- | --- | --- | --- | --- | --- | --- |
|  | **1 dpi** | **2 dpi** | **3 dpi** | **4 dpi** | **5 dpi** | **6 dpi** | **7 dpi*** | **8 dpi** |
| Control | 0/10 | 0/10 | 0/5 | 0/5 | 0/5 | 0/5 | 0/5 | - |
| wtFAdV-4 | 10/10 | 10/10 | 1/1 | 1/1 | 1/1 | 0/1 | 0/1 | - |
| rFAdV-4 | 10/10 | 10/10 | - | - | - | - | - | - |
| rON1 | 0/10 | 0/10 | 0/5 | 0/5 | 0/5 | 0/5 | 0/5 | - |
| **Number of the organs with gross lesions at 2 dpi** | | | | | | | | |
| **Lesions** | **Control** | | **wtFAdV-4** | | **rFAdV-4** | | **rON1** | |
| Hydropericardium | 0/5 | | 4/5 | | 4/5 | | 0/5 | |
| Yellowish liver | 0/5 | | 5/5 | | 5/5 | | 0/5 | |
| Congested spleen | 0/5 | | 5/5 | | 5/5 | | 0/5 | |
| Swollen kidney | 0/5 | | 5/5 | | 4/5 | | 0/5 | |
| **Number of the organs with histopathological changes at 2 dpi** | | | | | | | | |
| **Organs** | **Control** | | **wtFAdV-4** | | **rFAdV-4** | | **rON1** | |
| Liver | 0/5 | | 5/5 | | 5/5 | | 1/5 | |
| Spleen | 0/5 | | 5/5 | | 5/5 | | 3/5 | |
| Kidney | 0/5 | | 5/5 | | 5/5 | | 2/5 | |

**Note:** The symbol ‘-’ indicates all chickens had died at this time point, and the symbol ‘*’ indicates the day ending the animal trials.
